# Ca_v_β surface charged residues contribute to the regulation of neuronal calcium channels

**DOI:** 10.1186/s13041-021-00887-3

**Published:** 2022-01-03

**Authors:** Alexandra Tran-Van-Minh, Michel De Waard, Norbert Weiss

**Affiliations:** 1 The Francis Crick Institute, London, Great Britain; 2 Inserm, L’Institut du Thorax, Université de Nantes, CHU Nantes, CNRS, Nantes, France; 3 LabEx Ion Channels, Science and Therapeutics, Valbonne, France; 4 Department of Pathophysiology, Third Faculty of Medicine, Charles University, Prague, Czech Republic; 5 Institute of Organic Chemistry and Biochemistry, Czech Academy of Sciences, Prague, Czech Republic; 6 Institute of Biology and Medical Genetics, First Faculty of Medicine, Charles University, Prague, Czech Republic; 7 Center of Biosciences, Institute of Molecular Physiology and Genetics, Slovak Academy of Sciences, Bratislava, Slovakia

**Keywords:** Ion channels, Calcium channels, Voltage-gated calcium channels, Ca_v_2.1 channels, Ca_v_β subunit, Alanine-scanning mutagenesis

## Abstract

Voltage-gated calcium channels are essential regulators of brain function where they support depolarization-induced calcium entry into neurons. They consist of a pore-forming subunit (Ca_v_α_1_) that requires co-assembly with ancillary subunits to ensure proper functioning of the channel. Among these ancillary subunits, the Ca_v_β plays an essential role in regulating surface expression and gating of the channels. This regulation requires the direct binding of Ca_v_β onto Ca_v_α_1_ and is mediated by the alpha interacting domain (AID) within the Ca_v_α_1_ subunit and the α binding pocket (ABP) within the Ca_v_β subunit. However, additional interactions between Ca_v_α_1_ and Ca_v_β have been proposed. In this study, we analyzed the importance of Ca_v_β_3_ surface charged residues in the regulation of Ca_v_2.1 channels. Using alanine-scanning mutagenesis combined with electrophysiological recordings we identified several amino acids within the Ca_v_β_3_ subunit that contribute to the gating of the channel. These findings add to the notion that additional contacts besides the main AID/ABP interaction may occur to fine-tune the expression and properties of the channel.

**Supplementary Information:**

The online version contains supplementary material available at 10.1186/s13041-021-00887-3.

## Main text

**Fig. 1:**
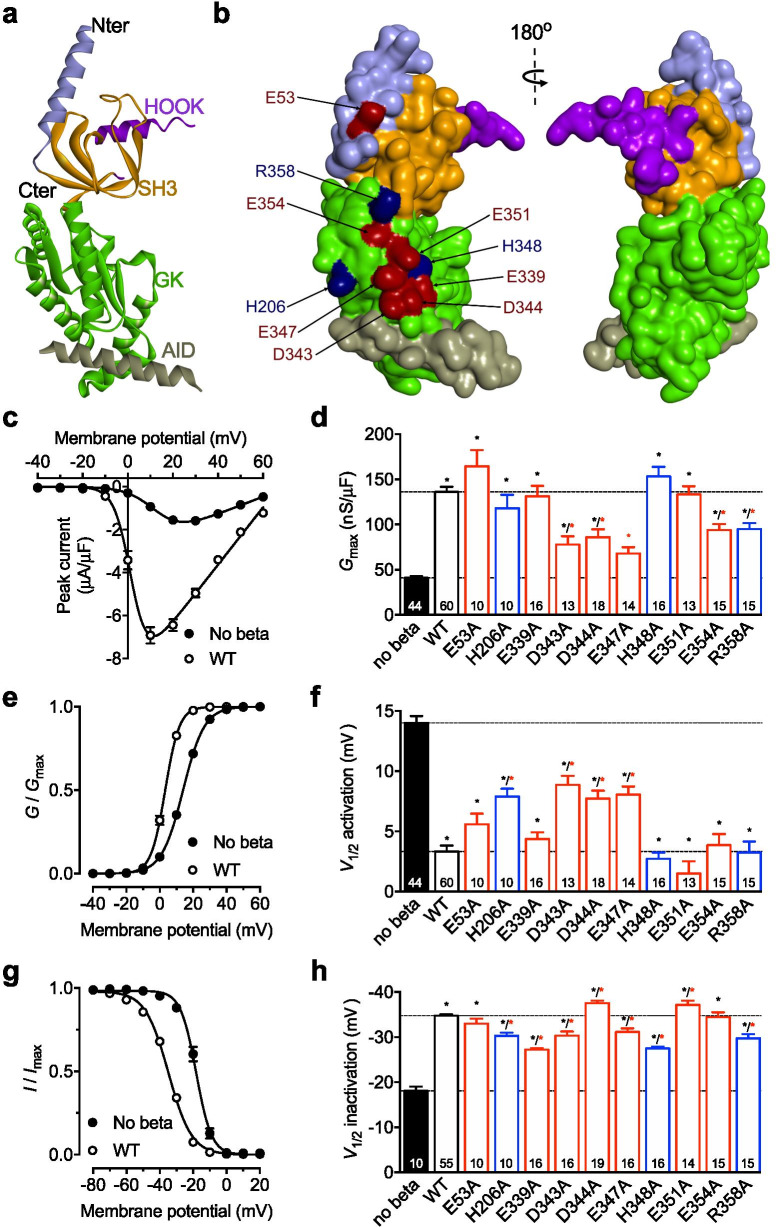
Ca_v_β_3_ surface charged residues contribute to the modulation of Ca_v_2.1 channels. **a** Cartoon representation of secondary structural elements of the rat Ca_v_β_3_ subunit in complex with the Ca_v_β_1_ interacting domain (AID) (PDB 1VYT). **b** Position of surface charged residues within the Ca_v_β_3_ subunit. Positively (H, histidine; R, arginine) and negatively (E, glutamic acid; D, aspartic acid) charged residues are shown in blue and red, respectively. **c** Mean current–voltage (*I*/*V*) relationship for Ca_v_2.1 expressed alone (filled circles) and in combination with wild-type Ca_v_β_3_ (open circles). **d** Corresponding mean maximal macroscopic conductance (*G*_max_) obtained from the fit of the *I*/*V* curves with the modified Boltzmann function (1) for Ca_v_2.1 alone and in combination with WT and mutant Ca_v_β_3_ (ANOVA results: F = 26.6; p < 0.0001 and F = 10.15; p < 0.0001 for Ca_v_2.1 expressed alone versus in the presence of Ca_v_β_3_ variants and Ca_v_2.1 expressed with Ca_v_β_3_ wild-type versus with Ca_v_β_3_ variants, respectively. **e** Mean normalized voltage-dependence of activation for Ca_v_2.1 expressed alone (filled circles) and in combination with WT Ca_v_β_3_ (open circles). **f** Corresponding mean half-activation potential values obtained from the fit of the activation curves with the modified Boltzmann function (1) for Ca_v_2.1 alone and in combination with WT and mutant Ca_v_β_3_ (ANOVA results: F = 34.29; p < 0.0001 and F = 9.965; p < 0.0001 for Ca_v_2.1 expressed alone versus in the presence of Ca_v_β_3_ variants and Ca_v_2.1 expressed with Ca_v_β_3_ wild-type versus with Ca_v_β_3_ variants, respectively). **g** Mean normalized voltage-dependence of inactivation for Ca_v_2.1 expressed alone (filled circles) and in combination with WT Ca_v_β_3_ (open circles). **h** Corresponding mean half-inactivation potential values obtained from the fit of the activation curves with the two-state Boltzmann function (3) for Ca_v_2.1 alone and in combination with WT and mutant Ca_v_β_3_ (ANOVA results: F = 47.9; p < 0.0001 and F = 27.84; p < 0.0001 for Ca_v_2.1 expressed alone versus in the presence of Ca_v_β_3_ variants and Ca_v_2.1 expressed with Ca_v_β_3_ wild-type versus with Ca_v_β_3_ variants, respectively. Statistical analysis (ANOVA followed by Dunnett’s post hoc multiple comparisons test) was performed for all Ca_v_β_3_ variants either against Ca_v_2.1 expressed alone (black statistical symbols) or Ca_v_2.1 expressed with WT Ca_v_β_3_ (red statistical symbols): * *p* < 0.05. The exact *p* values of the Dunnett’s post hoc analysis are provided in Additional file 3: Table S2

Neuronal high-voltage-activated (HVA) calcium channels are multisubunits complexes that support depolarization-induced calcium entry and downstream cellular functions [1]. They are composed of a pore-forming subunit (Ca_v_α_1_) that consists of four homologous membrane domains, each composed of six transmembrane helices, connected via cytoplasmic linkers (I–II, II–III, and III–IV loops), and cytoplasmic amino- and carboxy termini. They require the co-assembly with ancillary subunits to ensure the proper functioning of the channel. Among these ancillary subunits, the cytoplasmic Ca_v_β regulates several aspects of HVA channels including their gating properties and expression at the cell surface (for review see [2]). Ca_v_β subunits are encoded by four different genes (Ca_v_β_1–4_) and belong to the family of membrane-associated guanylate kinase (MAGUK). They consist of a conserved core region formed by Src homology 3 (SH3) and guanylate kinase (GK) domains connected by a HOOK region, flanked by non-conserved amino- and carboxy-termini (Fig. 1a). The molecular assembly of the Ca_v_α_1_/Ca_v_β complex relies on a conserved 18 residue sequence within the I–II loop of Ca_v_α_1_ called α_1_ interaction domain (AID) [3] that binds into a hydrophobic groove within the GK domain of Ca_v_β termed AID-binding pocket (ABP) [4–6] (Fig. 1a). This high-affinity interaction is critical for Ca_v_β-mediated enhancement of Ca_v_α_1_ surface expression and gating. Mutation of key residues within the ABP that weakens or abolishes AID-ABP interaction severely alters the functional influence of Ca_v_β [7]. Besides the AID/ABP interaction, additional low-affinity contacts between Ca_v_α_1_ and Ca_v_β that do not involve the ABP have been proposed to confer essential Ca_v_β modulatory properties [8–10]. In this study, we aimed to assess the functional importance of Ca_v_β surface charged residues in the regulation of Ca_v_2.1 channels. To do so, we generated a number of Ca_v_β_3_ mutants where surface charged residues, most belonging to the GK domain, were replaced with an alanine (Fig. 1b), and recombinant Ca_v_β_3_ were expressed in *Xenopus* oocytes with Ca_v_2.1 for electrophysiological analyses in the presence of 40 mM barium as charge carrier. Ca_v_β_3_ was chosen over Ca_v_β_4_ because it induces a more pronounced phenotype on Ca_v_2.1 with faster inactivation kinetics, and also because according to our experience, the association of Ca_v_β_3_ with Ca_v_2.1 in expression experiments is more complete than of Ca_v_β_4_ which would have made the interpretation of Ca_v_β_4_ variants slightly more difficult overall. As expected, the maximal macroscopic conductance (*G*_max_) in cells expressing Ca_v_2.1 was increased by 3.3-fold (*p* = 0.0001) in the presence of wild-type (WT) Ca_v_β_3_ compared to cells expressing Ca_v_2.1 alone (Fig. 1c, d, Additional file 1: Fig. S1, Additional file 2: Table S1). Similarly, all Ca_v_β_3_ variants, except the E347A mutant, produced a significant increase of Ca_v_2.1 conductance indicative of the proper expression of Ca_v_β_3_ mutants (Fig. 1d, Additional file 1: Fig. S1, Additional file 2: Table S1). However, Ca_v_β_3_-dependent potentiation of Ca_v_2.1 currents was significantly reduced when residues D343, D344, E347, E354 (located in the GK domain), and R358 (located in the N-terminus) were mutated (ranging from 1.4-fold reduction for Ca_v_β_3_ R358A to 2.0-fold reduction for Ca_v_β_3_ E347A compared to WT Ca_v_β_3_) (Fig. 1d, Additional file 1: Fig. S1, Additional file 2: Table S1). While the exact underlying mechanisms have not been further investigated in this study, this alteration is likely to have resulted from either a decreased surface expression of the channel, or from a decreased Ca_v_β-dependent potentiation of the single channel gating (channel open probability and latency to first channel opening). Consistent with the latest, we observed that while co-expression of WT Ca_v_β_3_ produced a 10.7 mV hyperpolarizing shift (*p* = 0.0001) of the mean-half activation potential of Ca_v_2.1, this effect was significantly reduced when the channel was co-expressed with Ca_v_β_3_ D343A, D344A, and E347A (Fig. 1e and f, Additional file 1: Fig. S2, Additional file 2: Table S1). In contrast, mutation of residues E354 and R358 did not alter Ca_v_β_3_-mediated hyperpolarization of the voltage-dependence of activation of Ca_v_2.1 suggesting that the effect of Ca_v_β_3_ mutants on Ca_v_2.1 conductance may have resulted from distinct gating alteration. In that respect, we note that while Ca_v_β_3_ H206A did not alter the maximal macroscopic conductance of Ca_v_2.1-expressing cells (Fig. 1d, Additional file 1: Fig. S1, Additional file 2: Table S1), it reduced the hyperpolarizing shift of the voltage-dependence of activation produced by WT Ca_v_β_3_ (Fig. 1f, Additional file 1: Fig. S2, Additional file 2: Table S1). Finally, we assessed the effect of Ca_v_β_3_ surface charged residues on the voltage-dependence of inactivation of the channel. Co-expression of WT Ca_v_β_3_ produced a 16.7 mV hyperpolarizing shift (*p* = 0.0001) of the mean-half inactivation potential of Ca_v_2.1 (Fig. 1g and h**, **Additional file 1: Fig. S3, Additional file 2: Table S1). Although this effect was significantly altered upon mutation of Ca_v_β_3_ surface charged residues, the magnitude of this alteration remained modest and all Ca_v_β_3_ mutants retained their ability to significantly enhance the voltage-dependence of inactivation of the channel (Fig. 1h, Additional file 1: Fig. S3, Additional file 2: Table S1). Indeed, the weakest enhancement was observed with Ca_v_β_3_ E339A and H348A which still produced a 9.1 mV 9.4 mV hyperpolarized shift, respectively, suggesting that Ca_v_β_3_ surface charged residues have minimal influence on the voltage-dependence of inactivation of Ca_v_2.1 channels. These data however allow us to conclude that for the Ca_v_β_3_ mutations for which there is a reduced *G*_max_ (Fig. 1d), the channels under study remain in the Ca_v_2.1 / Ca_v_β_3_ complex form.

While AID-ABP interaction is a prerequisite for Ca_v_β-dependent modulation of HVA channels, additional interactions are expected to contribute to Ca_v_β modulatory properties [8, 9]. Here, we reported that Ca_v_β surface charged residues located outside of the ABP play a significant role in Ca_v_β_3_-dependent modulation of Ca_v_2.1 channels. In particular, residues D343, D344, and E347 appear to form a hot-spot at the surface of the GK domain to influence activation of the channel, with limited effect on its inactivation. It is of interest that this cluster of residues is in close proximity to the AID sequence itself (Fig. 1B). These data are consistent with previous studies showing that the effect of Ca_v_β on the voltage-dependence of Ca_v_2.1 channel activation is largely reconstituted by the core region of Ca_v_β [11]. The question then arises as to how surface charged residues regulate channel gating. One possibility is via enabling additional low affinity interactions between Ca_v_β and other parts of Ca_v_α_1_. For instance, the amino- and carboxy-termini as well as the III–IV loop of Ca_v_α_1_ have been shown to interact directly with Ca_v_β [8, 9, 12, 13]. In addition, it was reported that the orientation of Ca_v_β relative to Ca_v_β_1_ is essential for Ca_v_β-mediated regulation of the channel activation [14, 15]. Therefore, it is a possibility that surface charged residues, by supporting low affinity interactions, may contribute to the proper positioning of Ca_v_β. Inherent to our study are a number of limitations that will need to be addressed in future studies. First, in addition to Ca_v_β, Ca_v_2.1 associated with Ca_v_α_2_δ that on the one hand mediates its own effects on the channel, and on the other hand influences the modulatory input of Ca_v_α_2_δ. For that reason, Ca_v_α_2_δ was purposely left out of our experiments to simplify the mechanistic analysis of Ca_v_β_3_ variants. However, given the important role of Ca_v_α_2_δ in the modulation of Ca_v_2.1, the present findings will need to be confirmed in the presence of Ca_v_α_2_δ where it can be expected that allosteric modulations will add another level of complexity to the regulation described in the present study. Second, in this study we used Ca_v_β_3_ because it produces a more pronounced phenotype on Ca_v_2.1 evidenced by faster inactivation kinetics compared for instance to Ca_v_β_4_, and also because according to our experience the associated of Ca_v_β_3_ with Ca_v_2.1 in expression experiments is more complete than of Ca_v_β_4_ which would have made the interpretation of the data more complicated. However, and although Ca_v_β_3_ represents a legitimate subunit that does associate with Ca_v_2.1 in native condition, Ca_v_β_4_ remains the major isoform found co-associated with Ca_v_2.1 in the brain and therefore it will be important to confirm our findings in the presence of Ca_v_β_4_. And third, another potential limitation inherent to our experimental settings is the use of *Xenopus* oocytes where trace levels of endogenous Ca_v_β have been reported. While such an endogenous Ca_v_β is unlikely to have played a major role in the regulation of recombinant Ca_v_2.1 since otherwise we would not have observed any effect of the co-expression of Ca_v_β_3_, it would nevertheless be important to reproduce these findings in a mammalian cell line.

## Supplementary Information

**Additional file 1: Fig. S1.** Effect of Ca_v_β_3_ mutants on Ca_v_2.1 current density. a Mean current–voltage (*I*/*V*) relationship for Ca_v_2.1 channels expressed alone (filled circles) and in the presence of wild-type (WT) Ca_v_β_3_ ancillary subunit (open circles). b–k Legend same as in (a) but for Ca_v_2.1 channels expressed with Ca_v_β_3_ mutants (open red circles). The smooth lines correspond to the fit of the *I*/*V* curve with the modified Boltzmann function (1). The dotted line shows the position of the *I*/*V* curve for Ca_v_2.1 expressed with WT Ca_v_β_3_ for comparison. **Fig. S2.** Effect of Ca_v_β_3_ mutants on the voltage-dependence of activation of Ca_v_2.1 channels. a Mean normalized voltage-dependence of activation for Ca_v_2.1 channels expressed alone (filled circles) and in the presence of wild-type (WT) Ca_v_β_3_ ancillary subunit (open circles). b–k Legend same as in (a) but for Ca_v_2.1 channels expressed with Ca_v_β_3_ mutants (open red circles). The smooth lines correspond to the fit of the activation curve with the modified Boltzmann function (2). The dotted line shows the voltage-dependence of activation for Ca_v_2.1 expressed with WT Ca_v_β_3_ for comparison. **Fig. S3.** Effect of Ca_v_β_3_ mutants on the voltage-dependence of inactivation of Ca_v_2.1 channels. a Mean normalized voltage-dependence of inactivation for Ca_v_2.1 channels expressed alone (filled circles) and in the presence of wild-type (WT) Ca_v_β_3_ ancillary subunit (open circles). b–k Legend same as in (a) but for Ca_v_2.1 channels expressed with Ca_v_β_3_ mutants (open red circles). The smooth lines correspond to the fit of the inactivation curve with the two-state Boltzmann function (3). The dotted line shows the voltage-dependence of inactivation for Ca_v_2.1 expressed with WT Ca_v_β_3_ for comparison.

**Additional file 2: Table S1.** Electrophysiological properties of Ca_v_2.1 channel expressed in *Xenopus* oocytes in the presence of Ca_v_β_3_ mutants. Statistical analysis (one-way ANOVA followed by Dunnett’s post hoc multiple comparisons test) was performed for all Ca_v_β_3_ variants against Ca_v_β_3_ wild-type (WT): *p < 0.05. β decreased conductance; β depolarized shift of voltage-dependence; β hyperpolarized shift of voltage-dependence.

**Additional file 3: Table S2.** Statistical summary. One-way analysis of variance (ANOVA) followed by Dunnett’s post hoc multiple comparisons test was used to determine statistical significance between Ca_v_β_3_ variants against channel expressed alone (top table) and against channel expressed with wild-type (WT) Ca_v_β_3_ (bottom table). Adjusted *p* values from Dunnett's multiple comparisons test are presented.

## Data Availability

All data generated or analyzed during this study are included in this published article and its supplementary information files.

## Abbreviations

ABP: AID-binding pocket
AID: Alpha interaction domain
GK: Guanylate kinase
HVA: High-voltage-activated channels
MAGUK: Membrane-associated guanylate kinase
SH3: Src homology 3
