# Supplementary material for: Cavβ surface charged residues contribute to the regulation of neuronal calcium channels
Source: Mol Brain. 2022 Jan 3;15:3. doi: 10.1186/s13041-021-00887-3 (PMC8722133; doi:10.1186/s13041-021-00887-3)
Supplement: Supplementary file 1 — Additional file 1: Fig. S1. Effect of Cavβ3 mutants on Cav2.1 current density. a Mean current–voltage (I/V) relationship for Cav2.1 channels expressed alone (filled circles) and in the presence of wild-type (WT) Cavβ3 ancillary subunit (open circles). b–k Legend same as in (a) but for Cav2.1 channels expressed with Cavβ3 mutants (open red circles). The smooth lines correspond to the fit of the I/V curve with the modified Boltzmann function (1). The dotted line shows the position of the I/V curve for Cav2.1 expressed with WT Cavβ3 for comparison. Fig. S2. Effect of Cavβ3 mutants on the voltage-dependence of activation of Cav2.1 channels. a Mean normalized voltage-dependence of activation for Cav2.1 channels expressed alone (filled circles) and in the presence of wild-type (WT) Cavβ3 ancillary subunit (open circles). b–k Legend same as in (a) but for Cav2.1 channels expressed with Cavβ3 mutants (open red circles). The smooth lines correspond to the fit of the activation curve with the modified Boltzmann function (2). The dotted line shows the voltage-dependence of activation for Cav2.1 expressed with WT Cavβ3 for comparison. Fig. S3. Effect of Cavβ3 mutants on the voltage-dependence of inactivation of Cav2.1 channels. a Mean normalized voltage-dependence of inactivation for Cav2.1 channels expressed alone (filled circles) and in the presence of wild-type (WT) Cavβ3 ancillary subunit (open circles). b–k Legend same as in (a) but for Cav2.1 channels expressed with Cavβ3 mutants (open red circles). The smooth lines correspond to the fit of the inactivation curve with the two-state Boltzmann function (3). The dotted line shows the voltage-dependence of inactivation for Cav2.1 expressed with WT Cavβ3 for comparison. [file 13041_2021_887_MOESM1_ESM.docx]

**Ca_v_β_3_ surface charged residues contribute to the regulation of neuronal Ca_v_2.1 calcium channels**

**Additional information**

**Additional methods**

**Molecular biology**

The cDNAs used in this study were rabbit Ca_v_2.1 (GenBank accession number M23919) and rat Ca_v_β_3_ (GenBank accession number M88751). Mutations of Ca_v_β_3_ were produced by site-directed mutagenesis PCR using mutagenic primers to replace charged residues by an alanine residue and final constructs were verified by sequencing of the coding region of the plasmid cDNAs.

**Transient expression in *Xenopus* oocytes**

Stage V and VI oocytes were surgically removed from anesthetized adult *Xenopus* *laevis* and treated for 2–3 h with 2 mg/ml collagenase type 1A (Sigma). Injection into the cytoplasm of cells was performed with 46 nL of cRNA mixture (0.3 μg/μL of Ca_v_2.1 + 0.1 μg/μL of Ca_v_β_3_) *in vitro* transcribed using the SP6 or T7 mMessage mMachine Kit (Ambion). Cells were incubated at 19°C in defined nutrient oocyte medium.

**Electrophysiological recordings**

After incubation for 2–4 days, macroscopic currents were recorded at room temperature (22–24°C) using a two- electrode voltage-clamp in a bathing medium containing (in millimolar): Ba(OH)_2_ 40, NaOH 50, KCl 3, HEPES 10, niflumic acid 0.5, pH 7.4 with methanesulfonic acid. Electrodes filled with (in millimolar) KCl 140, EGTA 10, and HEPES 10 (pH 7.2) had a resistance between 0.5 and 1 MΩ. Macroscopic currents were recorded using a Digidata 1322A and GeneClamp 500B amplifier (Axon Instruments). Acquisition and analyses were performed using the pClamp 8 software (Axon Instruments). Recordings were filtered at 2 kHz. Leak current subtraction was performed on-line by a P/4 procedure.

**Mathematical analysis**

The current-voltage relationship (*I*/*V*) curve was fitted with the following modified Boltzmann equation (1):

$$\left( 1 \right) I\left( V \right)= Gmax \frac{(V-Vrev)}{1+ \exp\frac{(V0.5-V)}{k}}$$

with *I*(*V*) being the peak current amplitude at the command potential *V*, *G*max the maximum conductance, *V*rev the reversal potential, *V*_0.5_ the half-activation potential, and *k* the slope factor. The voltage-dependence of the whole-cell conductance was calculated using the following modified Boltzmann equation (2):

$$\left( 2 \right) G\left( V \right)= \frac{Gmax}{1+ \exp\frac{(V0.5-V)}{k}}$$

with *G*(*V*) being the conductance at the command potential *V*. The voltage-dependence of the steady-state inactivation was fitted with the following two-state Boltzmann function (3):

$$\left( 3 \right) I\left( V \right)= \frac{Imax}{1+ \exp\frac{(V-V0.5)}{k}}$$

with *I*_max_ corresponding to the maximal peak current amplitude and *V*_0.5_ to the half-inactivation voltage.

**Statistical analysis**

Data values are presented as mean ± S.E.M for *n* measurements. Statistical analysis was performed using GraphPad Prism 7. One-way analysis of variance (ANOVA) followed by Dunnett’s post hoc multiple comparisons test was used to determine statistical significance. Datasets were considered significantly different for *p* ≤ 0.05 *.

**Additional Figures and Tables**

**
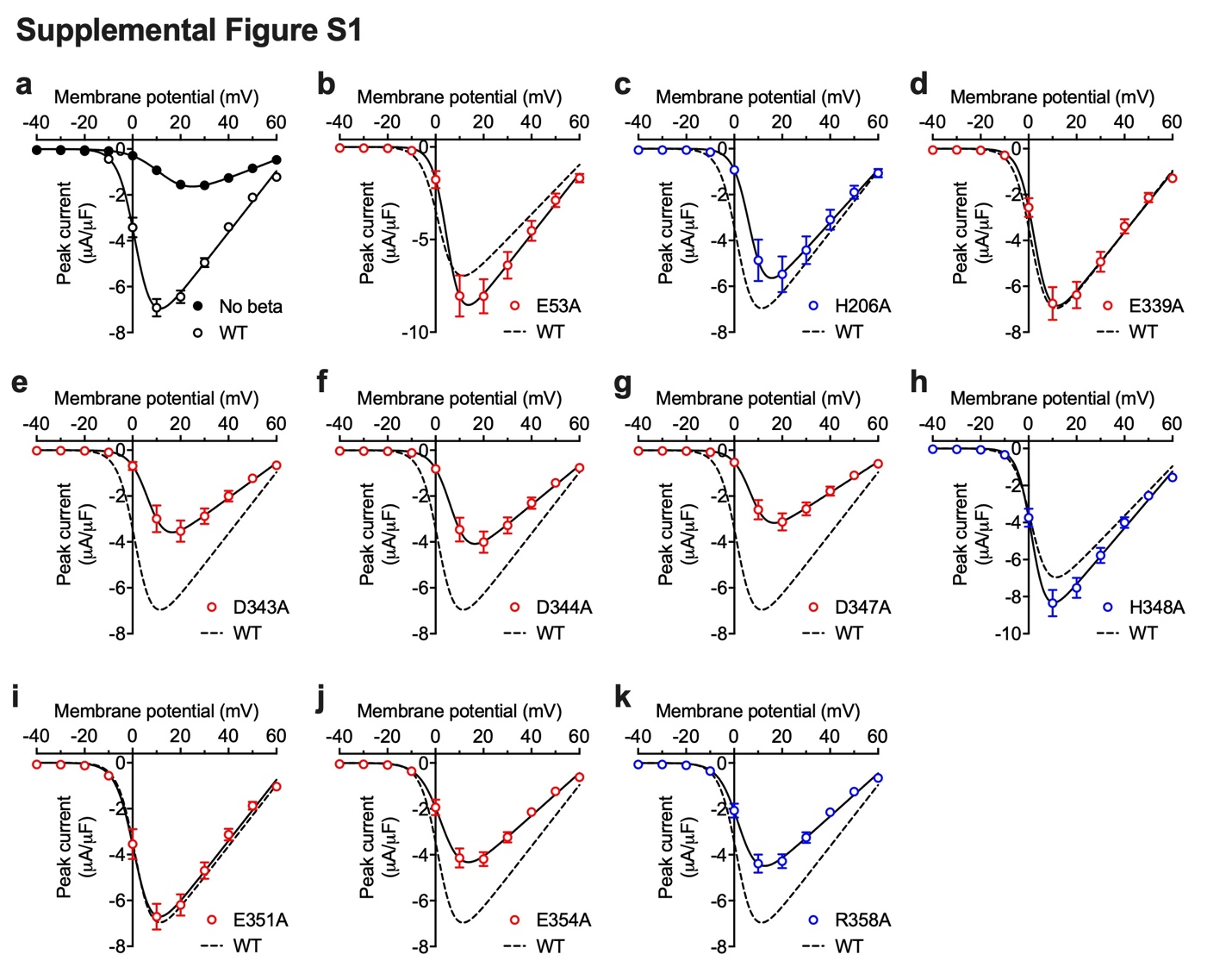
**

**Fig. S1** Effect of Ca_v_β_3_ mutants on Ca_v_2.1 current density. **a** Mean current-voltage (*I*/*V*) relationship for Ca_v_2.1 channels expressed alone (filled circles) and in the presence of wild-type (WT) Ca_v_β_3_ ancillary subunit (open circles). **b-k** Legend same as in **a** but for Ca_v_2.1 channels expressed with Ca_v_β_3_ mutants (open red circles). The smooth lines correspond to the fit of the *I*/*V* curve with the modified Boltzmann function (1). The dotted line shows the position of the *I*/*V* curve for Ca_v_2.1 expressed with WT Ca_v_β_3_ for comparison.


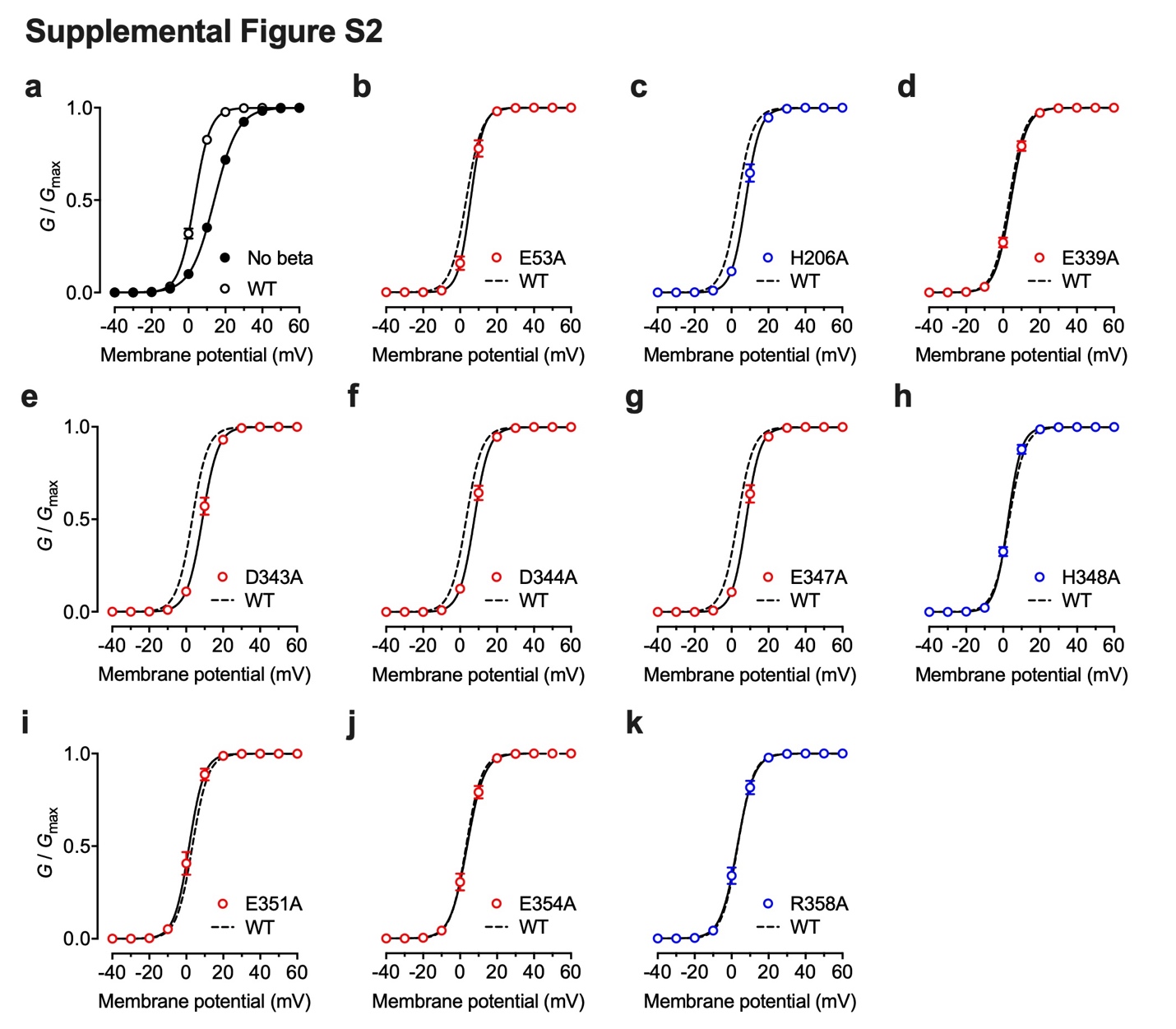


**Fig. S2** Effect of Ca_v_β_3_ mutants on the voltage-dependence of activation of Ca_v_2.1 channels. **a** Mean normalized voltage-dependence of activation for Ca_v_2.1 channels expressed alone (filled circles) and in the presence of wild-type (WT) Ca_v_β_3_ ancillary subunit (open circles). **b-k** Legend same as in **a** but for Ca_v_2.1 channels expressed with Ca_v_β_3_ mutants (open red circles). The smooth lines correspond to the fit of the activation curve with the modified Boltzmann function (2). The dotted line shows the voltage-dependence of activation for Ca_v_2.1 expressed with WT Ca_v_β_3_ for comparison.

**
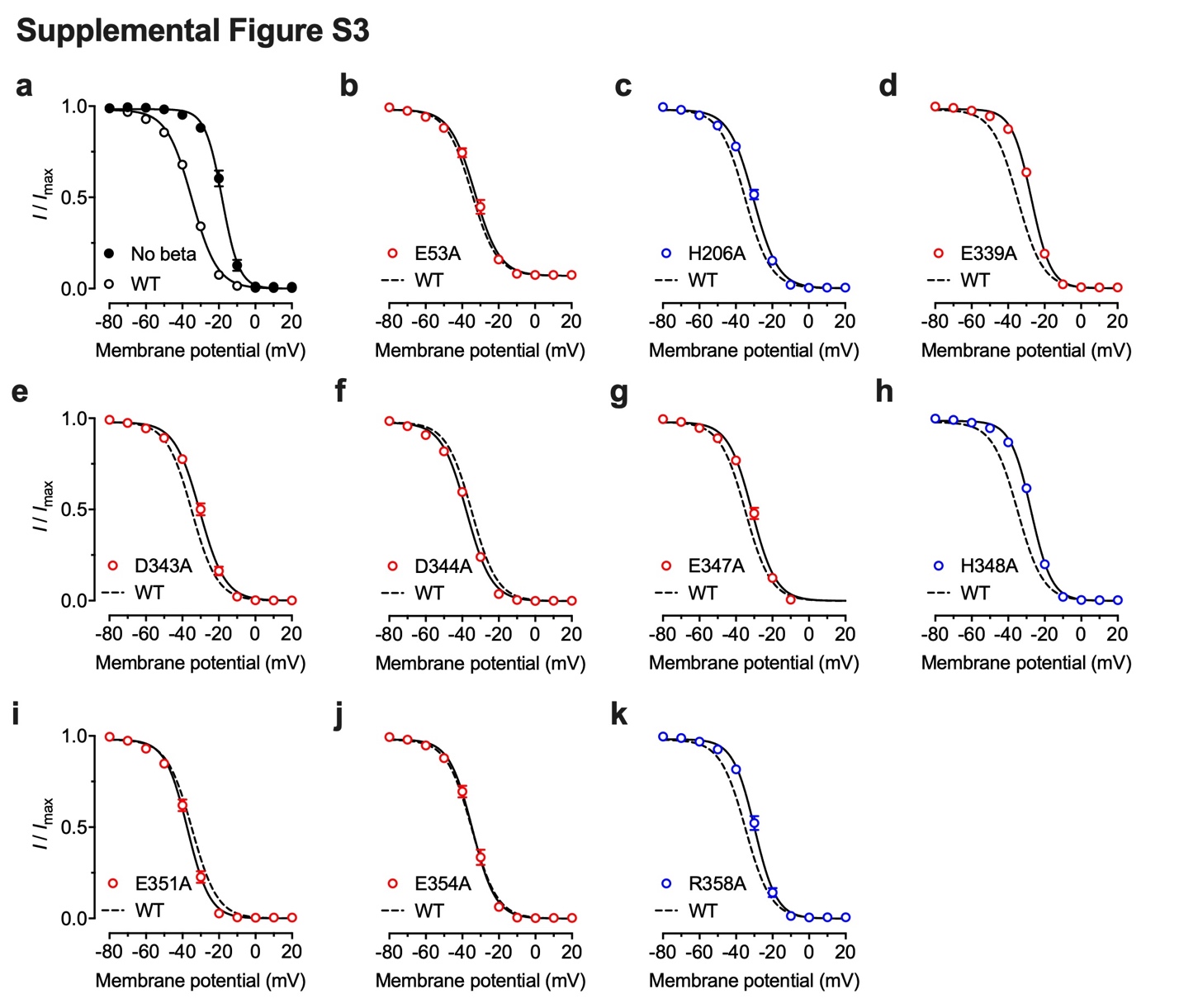
**

**Fig. S3** Effect of Ca_v_β_3_ mutants on the voltage-dependence of inactivation of Ca_v_2.1 channels. **a** Mean normalized voltage-dependence of inactivation for Ca_v_2.1 channels expressed alone (filled circles) and in the presence of wild-type (WT) Ca_v_β_3_ ancillary subunit (open circles). **b-k** Legend same as in **a** but for Ca_v_2.1 channels expressed with Ca_v_β_3_ mutants (open red circles). The smooth lines correspond to the fit of the inactivation curve with the two-state Boltzmann function (3). The dotted line shows the voltage-dependence of inactivation for Ca_v_2.1 expressed with WT Ca_v_β_3_ for comparison.
