# Supplementary material for: Cavβ surface charged residues contribute to the regulation of neuronal calcium channels
Source: Mol Brain. 2022 Jan 3;15:3. doi: 10.1186/s13041-021-00887-3 (PMC8722133; doi:10.1186/s13041-021-00887-3)
Supplement: Supplementary file 2 — Additional file 2: Table S1. Electrophysiological properties of Cav2.1 channel expressed in Xenopus oocytes in the presence of Cavβ3 mutants. Statistical analysis (one-way ANOVA followed by Dunnett’s post hoc multiple comparisons test) was performed for all Cavβ3 variants against Cavβ3 wild-type (WT): *p < 0.05. β decreased conductance; β depolarized shift of voltage-dependence; β hyperpolarized shift of voltage-dependence. [file 13041_2021_887_MOESM2_ESM.docx]

**Supplemental Table S1.** Electrophysiological properties of Ca_v_2.1 channel expressed in *Xenopus* oocytes in the presence of Ca_v_β_3_ mutants. Statistical analysis (one-way ANOVA followed by Dunnett’s post hoc multiple comparisons test) was performed for all Ca_v_β_3_ variants against Ca_v_β_3_ wild-type (WT): * *p* < 0.05. ↓ decreased conductance; ® depolarized shift of voltage-dependence; ¬ hyperpolarized shift of voltage-dependence.

|  |  | **Activation** | | | | | **Inactivation** | | |
| --- | --- | --- | --- | --- | --- | --- | --- | --- | --- |
| **Channel** | | ***G*_max_** (nA/μF) | | ***V*_0.5_** (mV) | | **(*n*)** | ***V*_0.5_** (mV) | | **(*n*)** |
| Ca_v_2.1 | | 40.8 ± 1.9 |  | 14.0 ± 0.6 |  | 44 | -18.1 ± 1.0 |  | 10 |
| +β_3_ | WT | 136.1 ± 5.6 |  | 3.3 ± 0.5 |  | 60 | -34.8 ± 0.3 |  | 55 |
|  | E53A | 164.4 ± 17.9 |  | 5.6 ± 0.9 |  | 10 | -33.0 ± 1.1 |  | 10 |
|  | H206A | 118.2 ± 14.7 |  | **7.9 ± 0.6*** | **®** | 10 | **-30.3 ± 0.7*** | **®** | 10 |
|  | E339A | 131.3 ± 11.5 |  | 4.4 ± 0.6 |  | 16 | **-27.2 ± 0.4*** | **®** | 16 |
|  | D343A | **77.8 ± 9.2*** | **↓** | **8.9 ± 0.8*** | **®** | 13 | **-30.3 ± 0.9*** | **®** | 16 |
|  | D344A | **85.9 ± 8.9*** | **↓** | **7.7 ± 0.7*** | **®** | 18 | **-37.6 ± 0.5*** | **¬** | 19 |
|  | E347A | **67.8 ± 7.1*** | **↓** | **8.1 ± 0.7*** | **®** | 14 | **-31.1 ± 0.8*** | **®** | 16 |
|  | H348A | 153.2 ± 10.5 |  | 2.7 ± 0.5 |  | 16 | **-27.5 ± 0.4*** | **®** | 16 |
|  | E351A | 133.3 ± 9.1 |  | 1.5 ± 1.0 |  | 13 | **-37.2 ± 0.9*** | **¬** | 14 |
|  | E354A | **93.8 ± 6.6*** | **↓** | 3.9 ± 0.9 |  | 15 | -34.4 ± 1.1 |  | 15 |
|  | R358A | **95.0 ± 6.6*** | **↓** | 3.2 ± 0.9 |  | 15 | **-29.8 ± 0.9*** | **®** | 15 |
