# Supplementary material for: Cavβ surface charged residues contribute to the regulation of neuronal calcium channels
Source: Mol Brain. 2022 Jan 3;15:3. doi: 10.1186/s13041-021-00887-3 (PMC8722133; doi:10.1186/s13041-021-00887-3)
Supplement: Supplementary file 3 — Additional file 3: Table S2. Statistical summary. One-way analysis of variance (ANOVA) followed by Dunnett’s post hoc multiple comparisons test was used to determine statistical significance between Cavβ3 variants against channel expressed alone (top table) and against channel expressed with wild-type (WT) Cavβ3 (bottom table). Adjusted p values from Dunnett's multiple comparisons test are presented. [file 13041_2021_887_MOESM3_ESM.docx]

**Supplemental Table S2.** Statistical summary. One-way analysis of variance (ANOVA) followed by Dunnett’s post hoc multiple comparisons test was used to determine statistical significance between Ca_v_β_3_ variants against channel expressed alone (top table) and against channel expressed with wild-type (WT) Ca_v_β_3_ (bottom table). Adjusted *p* values from Dunnett's multiple comparisons test are presented.

|  | ***G*_max_** | ***V*_0.5_ activation** | ***V*_0.5_ inactivation** |
| --- | --- | --- | --- |
| \| no beta vs. WT \| \| --- \| \| no beta vs. E53A \| \| no beta vs. H206A \| \| no beta vs. E339A \| \| no beta vs. D343A \| \| no beta vs. D344A \| \| no beta vs. E347A \| \| no beta vs. H348A \| \| no beta vs. E351A \| \| no beta vs. E354A \| \| no beta vs. R358A \| | \| 0.0001 \| \| --- \| \| 0.0001 \| \| 0.0001 \| \| 0.0001 \| \| 0.0123 \| \| 0.0001 \| \| 0.1134 \| \| 0.0001 \| \| 0.0001 \| \| 0.0001 \| \| 0.0001 \| | \| 0.0001 \| \| --- \| \| 0.0001 \| \| 0.0001 \| \| 0.0001 \| \| 0.0001 \| \| 0.0001 \| \| 0.0001 \| \| 0.0001 \| \| 0.0001 \| \| 0.0001 \| \| 0.0001 \| | \| 0.0001 \| \| --- \| \| 0.0001 \| \| 0.0001 \| \| 0.0001 \| \| 0.0001 \| \| 0.0001 \| \| 0.0001 \| \| 0.0001 \| \| 0.0001 \| \| 0.0001 \| \| 0.0001 \| |

|  | ***G*_max_** | ***V*_0.5_ activation** | ***V*_0.5_ inactivation** |
| --- | --- | --- | --- |
| \| WT vs. E53A \| \| --- \| \| WT vs. H206A \| \| WT vs. E339A \| \| WT vs. D343A \| \| WT vs. D344A \| \| WT vs. E347A \| \| WT vs. H348A \| \| WT vs. E351A \| \| WT vs. E354A \| \| WT vs. R358A \| | \| 0.2244 \| \| --- \| \| 0.7298 \| \| 0.9994 \| \| 0.0001 \| \| 0.0001 \| \| 0.0001 \| \| 0.5676 \| \| 0.9997 \| \| 0.0025 \| \| 0.0036 \| \|  \| | \| 0.2315 \| \| --- \| \| 0.0004 \| \| 0.8589 \| \| 0.0001 \| \| 0.0001 \| \| 0.0001 \| \| 0.9918 \| \| 0.3313 \| \| 0.9967 \| \| 0.9999 \| | \| 0.3860 \| \| --- \| \| 0.0001 \| \| 0.0001 \| \| 0.0001 \| \| 0.0023 \| \| 0.0001 \| \| 0.0001 \| \| 0.0374 \| \| 0.9994 \| \| 0.0001 \| |
